# Rhizobium aouanii sp. nov., efficient nodulating rhizobia isolated from Acacia saligna roots in Tunisia

**DOI:** 10.1099/ijsem.0.006515

**Published:** 2024-09-05

**Authors:** Jihed Hsouna, Takwa Gritli, Houda Ilahi, Jia-Cheng Han, Walid Ellouze, Xiao Xia Zhang, Maroua Mansouri, Praveen Rahi, Mustapha Missbah El Idrissi, Mouad Lamrabet, Pierre Emmanuel Courty, Daniel Wipf, Abdelkader Bekki, James T. Tambong, Bacem Mnasri

**Affiliations:** 1 Laboratory of Legumes and Sustainable Agrosystems, Centre of Biotechnology of Borj-Cédria, BP 901 Hammam-lif 2050, Tunisia; 2 University of Carthage, Faculty of Sciences of Bizerte, Tunis, Tunisia; 3 Agricultural Cultural Collection of China, Institute of Agricultural Resources and Regional Planning, Chinese Academy of Agricultural Sciences, Beijing 100080, PR China; 4 Agriculture and Agri-Food Canada, 4902 Victoria Avenue North, Vineland Station, Ontario, L0R 2E0, Canada; 5 Institut Pasteur, Université Paris Cité, Biological Resource Center of Institut Pasteur (CRBIP), Paris, France; 6 Faculty of Sciences, Centre de Biotechnologies Végétale et Microbienne, Biodiversité et Environnement, Mohammed V University in Rabat, Rabat, Morocco; 7 Agroécologie, Institut Agro Dijon, CNRS, Univ. Bourgogne, INRAE, Univ. Bourgogne Franche-Comté, F-21000 Dijon, France; 8 Biotechnology of Rhizobia and Plant Breeding Laboratory, Department of Biotechnology, Faculty of Sciences, University of Oran1, Sénia, Algeria; 9 Agriculture and Agri-Food Canada, 960 Carling Avenue, Ottawa, Ontario, K1A 0C6, Canada

**Keywords:** *Acacia saligna*, nodules, *Rhizobium aouanii*, *Rhizobium leguminosarum* complex, rhizobia

## Abstract

Three bacterial strains, 1AS14I^T^, 1AS12I and 6AS6, isolated from root nodules of *Acacia saligna*, were characterized using a polyphasic approach. Phylogenetic analysis based on *rrs* sequences placed all three strains within the *Rhizobium leguminosarum* complex. Further phylogeny, based on 1 756 bp sequences of four concatenated housekeeping genes (*recA*, *atpD*, *glnII* and *gyrB*), revealed their distinction from known rhizobia species of the *R. leguminosarum* complex (Rlc), forming a distinct clade. The closest related species, identified as *Rhizobium laguerreae*, with a sequence identity of 96.4% based on concatenated *recA*-*atpD*-*glnII*-*gyrB* sequences. The type strain, 1AS14I^T^, showed average nucleotide identity (ANI) values of 94.9, 94.3 and 94.1% and DNA–DNA hybridization values of 56.1, 57.4 and 60.0% with the type strains of closest known species: *R. laguerreae*, *Rhizobium acaciae* and *‘Rhizobium indicum’,* respectively. Phylogenomic analyses using 81 up-to-date bacteria core genes and the Type (Strain) Genome Server pipeline further supported the uniqueness of strains 1AS14I^T^, 1AS12I and 6AS6. The relatedness of the novel strains to NCBI unclassified *Rhizobium* sp. (396 genomes) and metagenome-derived genomes showed ANI values from 76.7 to 94.8% with a species-level cut-off of 96%, suggesting that strains 1AS14I, 1AS12I and 6AS6 are a distinct lineage. Additionally, differentiation of strains 1AS14I^T^, 1AS12I and 6AS6 from their closest phylogenetic neighbours was achieved using phenotypic, physiological and fatty acid content analyses. Based on the genomic, phenotypic and biochemical data, we propose the establishment of a novel rhizobial species, *Rhizobium aouanii* sp. nov., with strain 1AS14I^T^ designated as the type strain (=DSM 113914^T^=LMG 33206^T^). This study contributes to the understanding of microbial diversity in nitrogen-fixing symbioses, specifically within *Acacia saligna* ecosystems in Tunisia.

## Introduction

*Rhizobium leguminosarum* is recognized as the primary nodulating species of the tribe Vicieae [1,3]. Saidi *et al*. [4] showed that *R. leguminosarum* includes at least two distinct species namely *R. leguminosarum* USDA2370^T^ and *R. laguerreae* FB206^T^. Young *et al*. [5] analysed 439 whole-genome sequences of bacterial strains previously identified in NCBI database as *R. leguminosarum* and concluded that the strains are too diverse to be classified as a single species and named the group the *R. leguminosarum* complex (Rlc). In addition, 18 genospecies (gsp) were defined plus seven single unique strains that have no close relatives within the Rlc [5]. Ten of the genospecies include the type strains of validly published species. Genospecies E includes *R. leguminosarum* USDA 2370^T^, gspR is *R*. *laguerreae* [4], gspG is *Rhizobium sophorae*, gspC is *Rhizobium ruizarguesonis* [6], gspI is ‘*Rhizobium indicum’* [7], gspS is *Rhizobium changzhiense* [8], gspA is *Rhizobium brockwellii*, gspB is *Rhizobium johnstonii*, gspD is *Rhizobium beringeri* [9] and gspJ is *Rhizobium acaciae* [10]. This classification suggests the presence of several other clades within the Rlc that equally deserved formal species designation due to their distinct genetic makeup.

Hsouna *et al*. [11] reported that *Acacia saligna* plants grown in Tunisian soils were nodulated by fast-growing organisms, suggesting two putative new species within the genus *Rhizobium*. The authors published a new species (*R. acaciae*) within the genus *Rhizobium*, represented by strains 1AS11^T^ 1AS12, and 1AS13 [10]. The other potential new lineage, comprising the three strains 1AS14I^T^, 1AS12I and 6AS6, remained undescribed. Analysis of 1734 bp concatenated sequences of *recA*, *atpD*, *glnII* and *gyrB* genes placed these three strains in a distinct clade within the *R. leguminosarum* complex (Rlc), with *R. laguerreae* being the closest described species [11]. Interestingly, based on the divergence of *nod*A/*nod*C sequences and host range nodulation patterns, a criterion previously used for symbiovar delineation [12], strains 1AS14I^T^, 1AS12I and 6AS6 together with strains of * R. acaciae* (1AS11^T^, 1AS12, and 1AS13) were classified as a new symbiovar named salignae [11]. The representative strain 1AS14I^T^ was found to nodulate *A. saligna*, *A. salicina, Leucaena leucocephala*, but not *Glycine max*, *Phaseolus vulgaris* or *Retama raetam*, highlighting its unique symbiotic properties [11]. Despite this, the exact taxonomic position of these three strains remained unclear. This study present comprehensive phenotypic, biochemical, genomic and phylogenomic data for strains 1AS14I^T^, 1AS12I and 6AS6, underscoring their distinctiveness and suggesting their classification as a new taxon for which the name *Rhizobium aouanii* sp. nov. is proposed.

## Methods

### Isolation and ecology

Three novel *Rhizobium* strains were isolated from the root nodules of *A. saligna* plants grown in Tunisia, following the method described by Vincent [13]. Strains 1AS14I^T^ and 1AS12I were obtained from Borj Cedria (36° 42′ 35.5″ N 10° 25′ 42″ E), located in northern Tunisia, while strain 6AS6 was isolated from Monastir (35° 40′ 55.6″ N 10° 19′ 59.9″ E), situated in the central region of Tunisia. Single colonies of the bacterial cultures were obtained by repetitively streaking on yeast mannitol agar (YMA), as previously described by Hsouna *et al*. [11]. The type strain 1AS14I^T^ was deposited in the BCCM/LMG Bacteria Collection, University of Ghent, Belgium (LMG 33206^T^) and in the DSMZ-German Collection of Microorganisms and Cell Cultures, Leibniz Institute (DSM 113914^T^).

### *rrs*, *atpD*, *recA*, *glnII* and *gyrB* phylogeny

DNA extraction from strains 1AS14I^T^, 1AS12I and 6AS6 was performed following the method described by Terefework *et al*. [14]. The 16S rRNA gene sequence (1245 bp) and partial sequences of the *atpD*, *recA*, *glnII* and *gyrB* genes were amplified as previously described by Hsouna *et al*. [10]. PCR-amplified products were purified from agarose gels using the GeneJET Gel Extraction Kit (ThermoScientific) following the manufacturer’s instructions. Purified DNA fragments were sequenced at the Génome Québec Innovation Centre, Montreal, Canada. Sequences of the 16S rRNA, *atpD*, *recA*, *glnII* and *gyrB* housekeeping genes were used for phylogenetic analyses. Sequence similarities in the DNA databases were performed using the blast program (http://blast.ncbi.nlm.nih.gov/Blast.cgi). Reference sequences of 16S rRNA and the housekeeping genes were obtained from Genbank (NCBI). Sequences were aligned using the ClustalW2 software (http://www.ebi.ac.uk/Tools/clustalw2/). Phylogenetic trees were reconstructed using the mega 10 software package by the maximum-likelihood, neighbour-joining and minimum-evolution statistical methods [15], using the Kimura two-parameter substitution model [16]. Bootstrap values were based on 1000 replications and values >50% are shown on the phylogenetic trees.

### Genomic relatedness and phylogenomics

Draft whole-genome sequences were generated using Illumina NovaSeq 6000 (for strains 1AS14I^T^ and 1AS12I) or MiSeq (for strain 6AS6) technology (Génome Québec, Montreal, Canada). The quality of the raw reads [2×150 bp (NovaSeq) or 2×250 (MiSeq)] was checked using the FastQC algorithm [17]. Whole-genome sequence reads were assembled using Unicycler [18] and contigs <300 bp long were discarded, as implemented in BV-BRC pipeline [19]. The quality and completeness of the genome were evaluated using CheckM [20]. Annotations and protein function assignments were implemented in BV-BRC [19] or Prokka [21]. Average nucleotide identity (ANI [22]) and digital DNA–DNA hybridization (dDDH [23]) values were computed through pairwise comparisons between 1AS14I^T^ and type strains of closest related *Rhizobium* species. Phylogenomic analyses were done using Type (Strain) Genome Server (TyGS) [24]. The up-to-date bacterial core gene set pipeline of 81 single-copy genes (UBCG2 [25]) was used to validate the clustering patterns of the genome sequences and strains of *Rhizobium aouanii* sp. nov. identified on the TyGS-derived evolutionary tree. The UBCG-based tree was generated using RAxML-NG [26] with 1000 non-parametric bootstrap replicates. In addition, the type strain of *R. aouanii* sp. nov., strain 1AS14I^T^, was compared to genome sequences retrieved from GenBank as unclassified *Rhizobium* sp. (396 genomes) or uncultured metagenome-derived *Rhizobium* sp. (55 genomes) using ANI, dDDH and phylogenomics as described above.

In addition, we implemented the GTDB-Tk pipeline version 2.4.0 ([27] https://github.com/Ecogenomics/GTDBTk) to compare genome sequences of *R. aouanii* sp. nov. against the most recent GTDB database (release220) [28] consisting of over 12 000 bacterial and archaeal genomes from isolates and metagenomes derived from different environments. The classify_wf function of GTDB-Tk was used, which implements a workflow consisting of ANI screening with skani [29], Prodigal [30] with HMM models [31], and HMMER [32] to identify 120 bacterial and 53 archaeal marker genes, and pplacer [33 ] to find the maximum-likelihood placement of each genome in the GTDB-TK reference tree. FastTree [34] was used to infer the approximately maximum-likelihood trees for large alignments.

### Physiology and chemotaxonomy

Colony morphology was examined on YMA medium, and the phenotypic and physiological features of the three strains were determined and compared with their most related type strains: *R. acaciae* 1AS11^T^, *‘R. indicum’* JKLM 12A2^T^, and *R. laguerreae* FB206^T^. Characteristics tested included carbon sources utilization, tolerance to NaCl, pH, resistance to antibiotics, enzymatic activities, and fatty methyl ester content. Various phenotypic tests, including 70 carbon source utilization and 18 chemical sensitivity assays, were carried out using Biolog GEN III MicroPlates following the manufacturer's instructions. Enzymatic activities were assessed using the API ZYM systems (07584D and 25 200, bioMérieux). Antibiotic resistance testing was checked on Yeast Extract Mannitol (YEM) plates supplemented with five concentrations of each antibiotic: streptomycin, kanamycin, neomycin, and erythromycin. Tolerance to NaCl, pH, and temperature was monitored in liquid YEM medium as previously reported by Hsouna *et al*. [10]. The optimum pH for growth was assessed on YMA medium plates adjusted from pH 4 to 10 using 1 M HCl (pH 4.0 and 5.0) or 1 M NaOH (pH 7 to 10 prior to autoclaving). Fatty acid methyl esters analysis was performed using the Sherlock Microbial Identification System version 6.1 (midi) and a TSBA6 database.

## Results and discussion

### *rrs*, *atpD*, *recA*, *gln*II and *gyrB* phylogeny

**Fig. 1.**
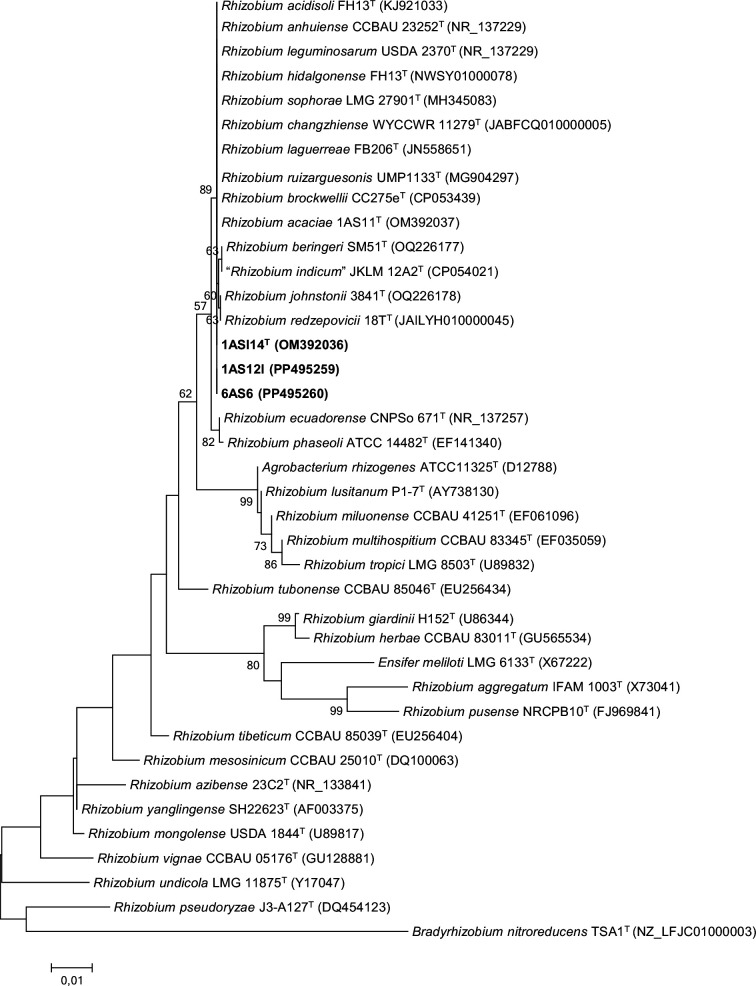
Maximum-likelihood-based phylogenetic tree of *rrs* sequences (1215 nucleotides) showing the *Rhizobium aouanii* strains clustering within the *Rhizobium leguminosarum* complex. The three novel strains are in bold. Bootstrap values ≥50 are indicated at the node (1000 replicates). After each species name, the strain code followed by the NCBI accession number are provided. The scale indicates the number of substitutions per site.

**Fig. 2.**
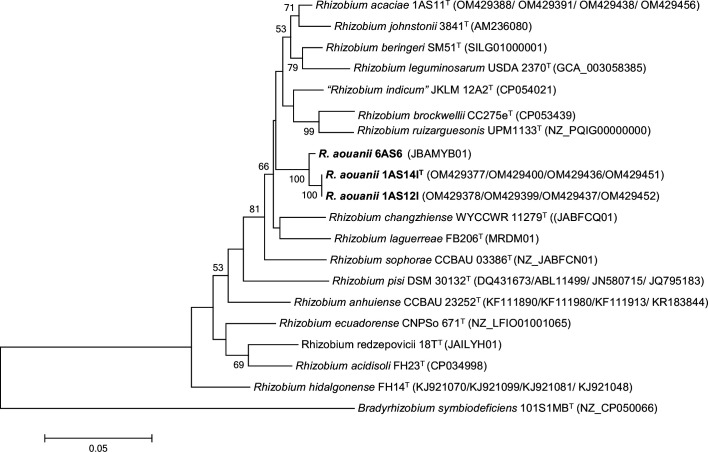
Maximum-likelihood-based evolutionary tree inferred from the concatenated *atpD-recA-glnII-gyrB* sequences (1734 nt). The three novel strains are in bold. Bootstrap values ≥50 are indicated for each node (1000 replicates). The scale indicates the number of substitutions per site.

The phylogenetic analysis based on partial *rrs* gene sequences (1215 nt) revealed that all three new strains clustered together, showing 100% similarity to type strains of *R. laguerreae*, *R. changzhiense*, *R. anhuiense*, *R. leguminosarum*, *R. ruizarguesonis*, * R. acidisoli*, *R. hidalgonense*, *‘R. indicum’*, *R. sophorae*, *R. redzepovicii*, *R. acaciae*, *R. brockwellii*, *R. johnstonii* and *R. beringeri*. These species are classified within the Rlc, with the exceptions of *R. anhuiense*, *R. acidisoli*, *R. hidalgonense*, and *R. redzepovicii*. The maximum-likelihood phylogenetic tree in Fig. 1 shows the clustering of the three strains of the proposed *R. aouanii* sp. nov. with the type strains of the Rlc. Similar tree topologies were obtained using the neighbour-joining (Fig. S1, available in the online Supplementary Material) and minimum-evolution (Fig. S2) algorithms. Given the limited taxonomic resolution at the species level obtained from the 16S rRNA gene sequences [35,36], multilocus sequence analysis of housekeeping genes was used to elucidate the *Rhizobium* species more precisely. Phylogenetic analysis based on concatenated *atpD-recA-glnII-gyrB* sequences (1734 bp) using the maximum-likelihood statistical method showed that the three strains of the proposed *R. aouanii* sp. nov formed a distinct cluster separate from the type strains of previously described *Rhizobium* species of the Rlc group (Fig. 2).

### Genomic relatedness and phylogenomics

Table 1 shows the basic statistics of the sequenced draft genomes. Strains 1AS14I^T^ and 1AS12I had slightly over 30 million raw NovaSeq reads, while strain 6AS6 had about 1.35 million raw MiSeq reads (Table 1). After quality checks, the reads used in the assembly process resulted in average coverage depths of 1376×, 1318× and 85×, with contig counts of 68, 67 and 58, for strains 1AS14I^T^, 1AS12I and 6AS6, respectively. The completeness of the genomes was 100%, with coarse and fine consistencies of 99.4 or 99.9 and 96.7%, respectively, and a contamination rate ranging from 0.2 to 0.5%. The N_50_ values were 288 843 (1AS14I^T^ or 1AS12I) and 314 702 (6AS6) bases, with an L_50_ of 9 for each strain (Table 1). The total length of the draft genomes of 1AS14I^T^, 1AS12I and 6AS6 were 7 110 981, 7 111 185 and 7 107 636 nucleotides, respectively, with a similar G+C content of 60.93 mol% (Table 1). A total of 4446 (1AS12I) or 4451 (1AS12I or 6AS6) protein-encoding genes with functional assignment were identified with 44 tRNA, three rRNA and one tmRNA each (Table 1). The newly generated draft whole-genome sequences were used to confirm the uniqueness of these strains relative to the closest related *Rhizobium* species.

**Table 1.** Whole-genome sequence statistics of the three strains of *Rhizobium aouanii* sp. nov. sequenced using Illumina NovaSeq 6000 or MiSeq technology

Description	1AS14I^T^	1AS12I	6AS6*
GenBank accession	JBAMYD01	JBAMYC01	JBAMYB01
Number of short paired end reads (151 or 250 bp length)	33112583	31707554	1349589
Average coverage depth	1376.8×	1318.7×	85.65×
Contig count	68	67	58
Largest contig (bp)	709429	709429	709429
Total length (bp)	7110981	7111185	7107636
Contigs N_50_ (bp)	288843	288843	314702
Contigs L_50_	9	9	8
Protein-encoding genes with functional assignment	4446	4451	4451
Total protein-coding sequences (CDSs)	7289	7294	7300
ncRNAs	4	4	4
rRNA (5S, 16S, 23S)	1, 1, 1	1, 1, 1	1, 1, 1
Number of tRNA	44	44	44
Number of tmRNA	1	1	1
G+C content (mol%)	60.93	60.93	60.93

Genome-based dDDH and ANI values were computed and compared to eight validly published close *Rhizobium* species to validate the taxonomic position of the strains. The three strains (1AS14I^T^, 1AS12I and 6AS6) exhibited pairwise dDDH and ANI values of 99.7 and 100%, respectively. These values are significantly above the species-level cut-offs of 70 and 96%, respectively, confirming their classification within the same *Rhizobium* species. Strain 1AS14I^T^ exhibited low dDDH and ANI values, falling below species delineation cut-off of 70 and 96%, respectively, with known *Rhizobium* species (Table 2). The closest *Rhizobium* species identified were ‘*R. indicum’* JKLM 12A2^T^, *R. acaciae* 1AS11^T^ and *R. laguerreae* FB206^T^ (Table 2). For example, ‘*R. indicum’* JKLM 12A2^T^ showed the highest dDDH of 60.0% (cut-off of 70%) and ANI of 94.95% (cut-off of 96.0%) with strain 1AS14I^T^ followed by *R. acaciae* 1AS11^T^ with values of 57.4 and 94.08% (Table 2). These findings confirmed the results of the *recA-atpD-gyrB-glnII* phylogenetic analysis, suggesting that strains 1AS14I^T^, 1AS12I and 6AS6 represent a novel species within the genus *Rhizobium*.

**Table 2.** Average nucleotide identity (ANI) and digital DNA–DNA hybridization (dDDH) values among *Rhizobium aouanii* sp. nov. 1AS14I^T^ (type strain) and related *Rhizobium* species

*Rhizobium* species	ANI (%)	dDDH (%)
*Rhizobium aouanii* sp. nov. 1AS14I^T^ (JBAMYD000000000)	100	100
*‘Rhizobium indicum’* JKLM 12A2^T^ (CP054021)	94.93	60.0
*Rhizobium acaciae* 1AS11^T^ (NZ_JAPCZO000000000)	94.30	57.4
*Rhizobium laguerreae* FB206^T^ (MRDM00000000)	94.07	56.1
*Rhizobium changzhiense* WYCCWR 11279^T^ (JABFCQ000000000)	93.73	53.7
*Rhizobium sophorae* CCBAU 03386^T^ (JABFCN000000000)	93.65	53.2
*Rhizobium ruizarguesonis* UPM 1133^T^ (PQIG00000000)	93.48	52.7
*Rhizobium leguminosarum* DSM 106839^T^ (JACHFX000000000)	92.71	49.1

**Fig. 3.**
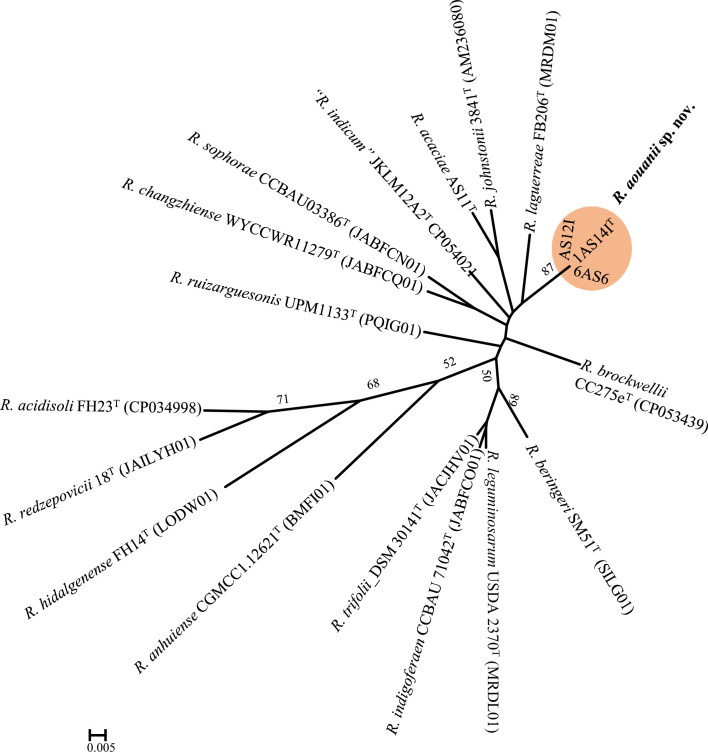
Distinct clustering of *Rhizobium aouanii* sp. nov. strains relative to their closest known taxa on an unrooted maximum likelihood phylogenomic tree based on 81 up-to-date bacterial core genes (UBCG2; Kim *et al*. [25]), inferred using RAxML-ng with the GTR +CAT model. Percentage bootstrap values >50% are indicated at branching points. Bar, 0.005 substitution per position.

The dDDH and ANI results were further supported by phylogenomics tree inferred using theTyGS [24] based on Genome blast Distance Phylogeny (Fig. S4). In addition, the uniqueness of the strains 1AS14I^T^, 1AS12I and 6AS6 was validated by the 81 UBCG pipeline [25] (Fig. 3). Both TyGS and UBCG generated phylogenomic trees showing that strains 1AS14I^T^, 1AS12I and 6AS6 clustered distinctly from known species of the genus *Rhizobium* (Figs 3 and S3). These genome data and analyses strongly indicate that these strains represent a potential new species in the genus *Rhizobium*.

**Fig. 4.**
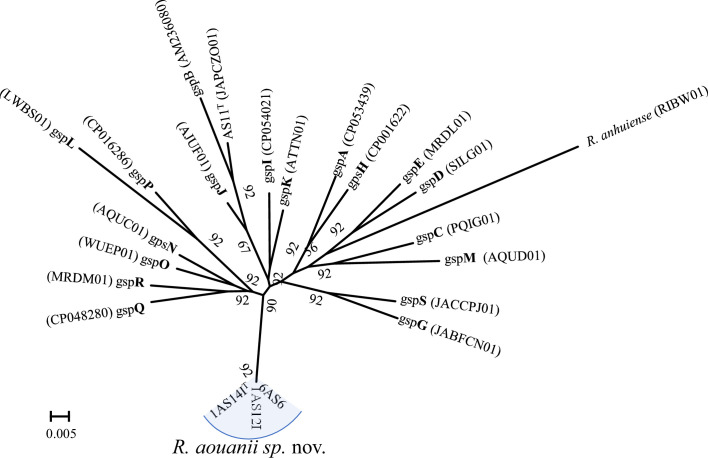
Up-to-date bacterial core genes (UBCG) maximum-likelihood-based phylogenetic tree of strains of *Rhizobium aouanii* sp. nov. and representatives of the 18 genospecies proposed (letters in bold) by Young *et al*. [5] suggests a new genospecies. The tree was inferred using RAxML-ng with the GTR +CAT model, and percentage bootstrap values are given at branching points.

Additionally, the genome data obtained in this study were utilized to investigate the strains' relatedness to the proposed 18 genospecies groupings (gsp) of Young *et al*. [5]. A maximum-likelihood phylogenomics tree generated using the UBCG tool showed strains 1AS14I^T^, 1AS12I and 6AS6 clustering distinctly from the representative genomes of the 18 genospecies proposed by Young *et al*. [5] (Fig. 4). This tree suggests that the *R. aouanii* sp. nov. strains constitute a unique genospecies. Furthermore, Young *et al*. [5] identified seven unique strains (WSM1689, CCBAU10279, CC278f, Norway, WYCCWR10014, Tri-43 and Vaf12) that could not be associated to any of the defined 18 genospecies. A comparison of the genomes of these seven unique strains to *R. aouanii* sp. nov. strains revealed them to be highly divergent. For example, significantly low dDDH values ranging 48.20–60.5% were recorded between the seven strains and the type strain of *R. aouanii* sp. nov., 1AS14I^T^. These values which are significantly below the species delineation threshold of 70% suggest these strains represent distinct potential species that significantly differ from members of *R. aouanii* sp. nov.

**Fig. 5.**
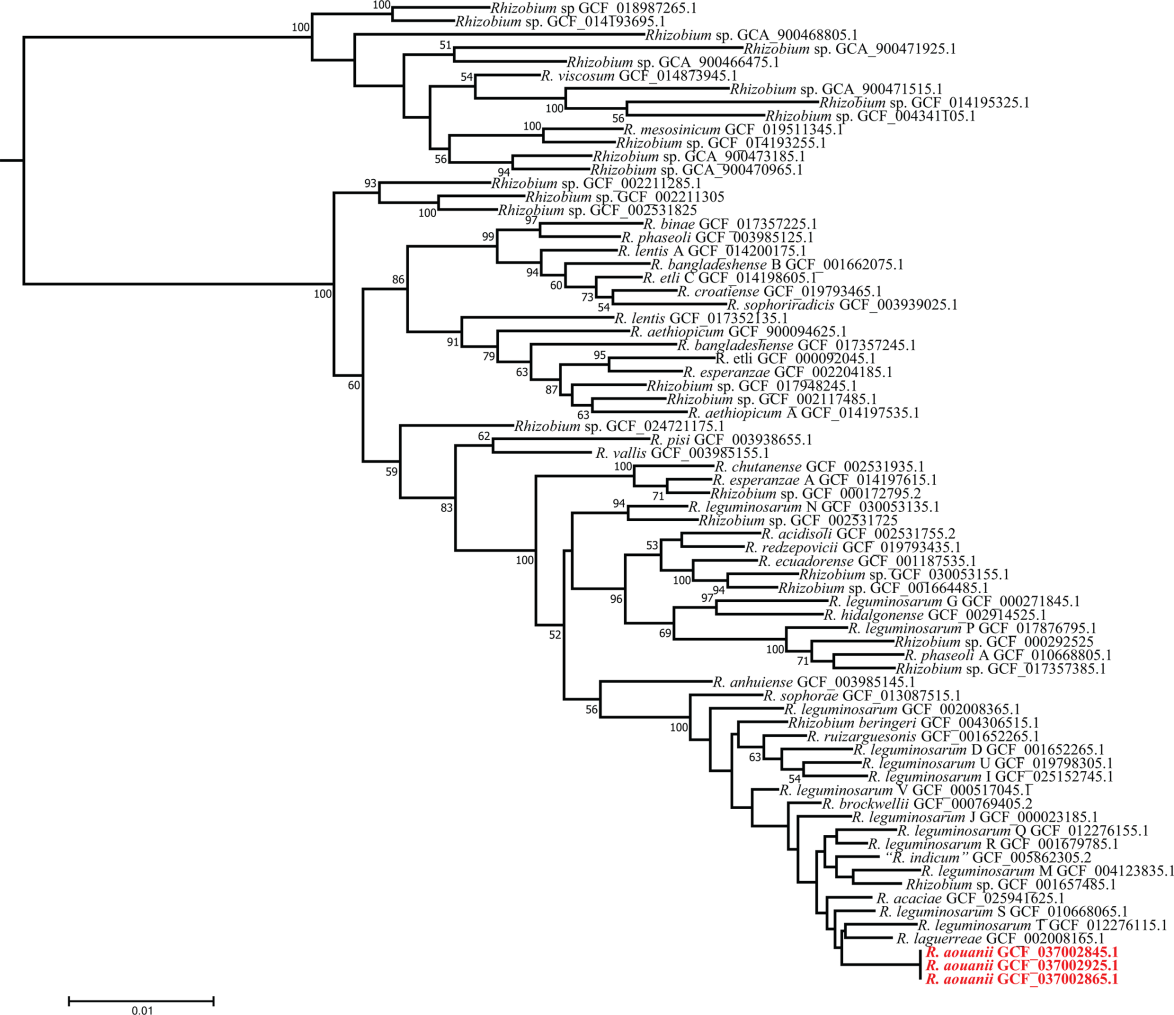
Subtree of GTDB-TK Bac120 protein set showing relatedness of strains of *Rhizobium aouanii* sp. nov. (in red) and the 70 whole-genome sequences of members of the *Rhizobium* group III generated using the classify_wf command and the most recent database (release220) of the Genome Taxonomy Database (GTDB-TK) pipeline version 2.4.0.

The uniqueness of the *R. aouanii* sp. nov. strains was further investigated using ANI values generated by Skani [29] and the BAC120 protein-alignment phylogenomic tree as implemented in the GTDB-TK pipeline. ANI values of 80.79–94.79% were obtained with *R. laguerreae* showing the highest value and *R. sullae* having the lowest value. *R. acaciae* [10], a strain isolated from Tunisia, exhibited the third highest ANI value of 94.41%. Fig. S4 shows a subtree of GTDB Bac120 protein set with *Rhizobium* genomes/strains in three distinct groupings (group I, group II and group III) well supported by high bootstrap values in relation with the close genera *Ectorhizobium*, *Agrobacterium*, *Neorhizobium* and *Allorhizobium*. Strains of *R. aouanii* sp. nov. clustered in group III together with 70 other *Rhizobium* strains and metagenome-derived genomes with *R. laguerreae* (=gspR) and a GTDB-defined taxon, s_*R. leguminosarum*_T (=gspQ), as the closest phylogenetic relatives (Fig. 5). This confirmed that strains 1AS14I, 6AS6 and 1AS12I constitute a novel lineage.

The *R. aouanii* sp. nov. strains were also evaluated against NCBI GenBank unclassified *Rhizobium* sp. (396 genomes) and the results show ANI values that are below the species-level cut-off and ranged from 76.7 to 94.8%, suggesting that strains 1AS14I^T^, 1AS12I and 6AS6 belong to a distinct lineage. We also investigated the relatedness between *R. aouanii* sp. nov. and metagenome-derived genomes. Of the 55 uncultured metagenome-derived *Rhizobium* sp. sequences, 15 were removed from the analysis due to potential contamination or had a genome size that is less than 4.0 Mb. The remaining 40 metagenome-derived genome sequences had ANI or dDDH values of 77.8–81.9% and 19.9–23.1% with strain 1AS14I^T^, which are below the species delineation threshold of 96% and 70%, respectively (Table S1). This affirmed the uniqueness of strains of the proposed *R. aouanii* sp. nov. The phylogenomic tree (Fig. S5) inferred using the 40 metagenome-derived genome sequences grouped the genomes into six distinct clusters. Cluster 1 regrouped the strains of *R. aouanii* sp. nov. with five metagenome-derived sequences: DAMCIK01 (urban metagenome, USA), CALLIZ01 (metagenome), JBBNAU01 (forest soil metagenome), DALZSY01 (urban metagenome) and SSFH01 (wastewater metagenome, USA) but the topology and branch lengths indicate significant genetic divergence. This is corroborated by low ANI and dDDH values (Table S1). Most of the metagenome-derived genomes analysed were obtained either in the USA or Europe with no entries from African countries including Tunisia. Also, the source materials, e.g. wastewater metagenome, of these MAG genomes are profoundly different from that of the strains of *R. aouanii* sp. nov. isolated from root nodules of * A. saligna* in Tunisia. Any similarities or differences between these taxa, however, may be overemphasized/overestimated given that metagenome-derived genome sequences are known to have quality and other issues including differences in genome sizes. For example, one metagenome-derived genome sequence deposited in GenBank as *Rhizobium* sp. has a genome size of 2.4 Mb instead of the required ca 7.1 Mb. A small genome may lack key genes required for bacterial classification. In fact, only 10 of these 40 metagenome-derived genomes had a copy of the 16S rRNA gene, a key genus-level marker. The metagenome-derived genome era has started but to effectively harness its usefulness in bacterial diversity and taxonomy, new sequencing methods to obtain very long reads coupled with smart bioinformatic tools to sort and assemble only fragments of the same molecule are key requirements.

### Physiology and chemotaxonomy

Colonies of strains 1AS14I^T^, 1AS12I and 6AS6 were typical rhizobial-like translucent, cream-white, and convex. These strains tolerated up to 0.8% NaCl, although none of them were able to grow at a concentration higher than 1%. All strains grew at pH levels from 5 to 10 and at temperatures ranging from 16 to 37 °C, with optimal growth at pH 7 and 28 °C. Carbon substrate utilization patterns (Table 3) shared among all three strains of *R. aouanii* sp. nov. differed from at least one of their closest related type strains. Particularly, these strains could assimilate methyl β-d-glucoside, d-fucose, l-rhamnose, d-arabitol, *myo*-inositol, glycerol, l-serine, β-hydroxy-d,l butyric acid, acetoacetic acid and acetic acid. However, they were unable to assimilate trehalose, gentiobiose, sucrose, turanose, stachyose, raffinose, *N*-acetyl-d-glucosamine, *N*-acetyl-β-d-mannosamine, α-d-glucose, d-mannose, d-glucose-6-PO_4_, d-fructose-6-PO_4_, l-pyroglutamic acid, d-galacturonic acid, d-gluconic acid, d-glucuronic acid, methyl pyruvate, l-malic acid and bromo-succinic acid. Other characteristics shown by the strains of the novel species that differed from at least one of these closest type strains were their positive enzymatic reactions for esterase, trypsin, acid phosphomonoesterase, β-galactosidase, α-galactosidase and *N*-acetyl-β-glucosaminase; and negative reaction for β-glucosidase. *Rhizobium aouanii* sp. nov. strains were resistant to lincomycin, 10 µg ml^−1^ kanamycin, 20 µgml^−1^ streptomycin and 20 µgml^−1^ neomycin but sensitive to 20 µgml^−1^ erythromycin. Analysis of fatty acid profiles revealed distinctive compositions, with notable differences in the amounts of certain fatty acids compared to related strains. The major fatty acids of the type strain 1AS14I^T^ were summed feature 8 (C_18 : 1_ ω7*c*; 48.68%), C_18 : 1_ ω7*c* 11-methyl (12.9%) and C_19 : 0_ cyclo ω8*c* (12.4%) (Table 4). The amount of C_18 : 1_ ω7*c* 11-methyl was higher than that in ‘*R. indicum’* JKLM 12A2^T^ and *R. laguerreae* FB206^T^ and the amount of C_19 : 0_ cyclo ω8*c* was higher than that in *R. acaciae* 1AS11^T^, *‘R. indicum’* JKLM 12A2^T^ and *Rhizobium laguerreae* FB206^T^. We also detected trace amounts of C_15 : 0_ 3OH (1.24%), C_17 : 1_ ω8*c* (1.06%), C_17 : 1_ ω6*c* (0.15%), C_17 : 0_ 3OH (1.24%), C_17 : 1_ anteiso ω9*c* (0.25%) and C_18 : 1_ ω9*c* (0.79%) that were absent in JKLM 12A2^T^ and FB206^T^.

**Table 3.** Phenotypic characteristics on Biolog GENIII MicroPlates and API ZYM for strains 1AS14I^T^, 1AS12I and 6AS6 of *Rhizobium aouanii* sp. nov. and related strains (1) ‘*Rhizobium indicum’* JKLM 12A2^T^, (2) *Rhizobium laguerreae* strain FB206^T^; (3) *Rhizobium acaciae* 1AS11^T^

Characteristic	*Rhizobium aouanii* sp.nov					
Biolog substrate	1AS14I^T^	1AS12I	6AS6	1	2	3
Trehalose	−	−	−	w	−	+
Gentiobiose	−	−	−	−	−	+
Sucrose	−	−	−	+	−	−
Turanose	−	−	−	w	−	+
Stachyose	−	−	−	−	−	+
Raffinose	−	−	−	−	−	+
Methyl β-d-glucoside	+	+	+	−	+	+
*N*-Acetyl-d-glucosamine	−	−	−	−	+	+
*N*-Acetyl-β-d-mannosamine	−	−	−	−	+	+
α-d-Glucose	−	−	−	−	−	+
d-Mannose	−	−	−	−	−	+
d-Fucose	+	+	+	−	+	+
l-Rhamnose	+	+	+	−	−	+
d-Arabitol	+	+	+	−	−	+
*myo*-Inositol	+	+	+	−	−	+
Glycerol	+	+	+	−	+	+
d-Glucose-6-PO_4_	−	−	−	−	+	−
d-Fructose-6-PO_4_	−	−	−	+	+	−
l-Pyroglutamic acid	−	−	−	−	+	+
l-Serine	+	+	+	−	−	−
d-Galacturonic acid	−	−	−	+	+	−
d-Gluconic acid	−	−	−	−	+	+
d-Glucuronic acid	−	−	−	+	+	−
Methyl pyruvate	−	−	−	−	−	+
l-Malic acid	−	+	+	−	+	−
Bromo-succinic acid	−	+	+	−	−	−
β-Hydroxy-d,l Butyric Acid	+	+	+	−	+	−
Acetoacetic acid	+	+	+	−	−	−
Acetic acid	+	+	+	w	−	−
**Chemical sensitivity**						
pH 6	+	+	+	−	−	+
pH 5	+	+	+	−	−	+
NaCl 1%	−	−	−	−	+	−
Tetrazolium violet	+	+	+	−	+	+
Tetrazolium blue	+	+	+	−	−	+
Potassium tellurite	+	+	+	−	−	+
**Antibiotic resistance**						
Lincomycin	+	+	+	−	−	−
Kanamycin (10 µg ml^−1^)	+	+	+		−	+
Erythromycin (20 µg ml^−1^)	−	−	−		w	w
Streptomycin (20 µg ml^−1^)	+	+	+		−	−
Neomycin (20 µg ml^−1^)	+	+	+		−	−
**API ZYM**						
Esterase	+	+	+	−	−	nd
Trypsin	+	+	+	w	w	nd
Acid phosphomonoesterase	+	+	+	−	−	nd
β-Galactosidase	+	+	+	w	w	nd
α-Galactosidase	+	+	+	+	−	nd
β-Glucosidase	−	−	−	w	+	nd
*N*-Acetyl-β-glucosaminase	+	+	+	−	−	nd

+, Positive; −, negative; w, weakly positive; nd, not determined.

**Table 4.** Fatty acid composition (%) of strain 1AS14I^T^ and related strains Strains: 1, 1AS14I^T^; 2, *Rhizobium acaciae* 1AS11^T^; 3, ‘*Rhizobium indicum’* JKLM 12A2^T^ ; 4, *Rhizobium laguerreae* FB206^T^. –, Not detected.

Fatty acid	1	2	3	4
C_15 : 0_ 3OH	1.24	0.27	–	–
C_17 : 0_	4.18	0.78	–	–
C_17 : 1_ ω8*c*	1.06	0.26	–	–
C_17 : 0_ 3OH	1.24	0.16	–	–
C_18 : 1_ ω7*c* 11-methyl	12.9	13.24	1.8	1.9
C_19 : 0_ cyclo ω8*c*	12.4	3.0	3.9	6.4
Summed feature 8 (C_18 : 1_ ω7*c*)	48.68	57.81	68	69.3

Based on the genotypic and phenotypic data presented in this study, we propose that strains 1AS14I^T^, 1AS12I and 6AS6 represent a novel species, named *Rhizobium aouanii* sp. nov.

## Description of *Rhizobium aouanii* sp. nov.

*Rhizobium aouanii* (a.ou.a’ni.i. N.L. gen. masc. n. *aouanii,* of Aouani, to honour Dr. Mohamed Elarbi Aouani, a Tunisian microbiologist who made significant contributions to research on rhizobia).

Cells of *Rhizobium aouanii* are Gram-stain-negative, aerobic, and non-spore-forming. When cultured on YMA medium, colonies appear translucent, cream-white and convex after 3 days of growth at 28 °C. The strains tolerated up to 0.8% NaCl but did not grow at concentrations >1%; and grew at pH levels from 5 to 10 and at temperatures ranging from 16 to 37 °C, with optimal growth at pH 7 and 28 °C. Strain 1AS14I^T^ demonstrated the ability to utilize various carbon sources, including methyl β-d-glucoside, d-fucose, l-rhamnose, d-arabitol, *myo*-inositol, glycerol, l-serine, β-hydroxy-d,l-butyric acid, acetoacetic acid and acetic acid; but is unable to use trehalose, gentiobiose, sucrose, turanose, stachyose, raffinose, *N*-acetyl-d-glucosamine, *N*-acetyl-β-d-mannosamine, α-d-glucose, d-mannose, d-glucose-6-PO_4_, d-fructose-6-PO_4_, l-pyroglutamic acid, d-galacturonic acid, d-gluconic acid, d-glucuronic acid, methyl pyruvate, l-malic acid, and bromo-succinic acid. Furthermore, strain 1AS14I^T^ is resistant to lincomycin, kanamycin (10 µg ml^−1^), streptomycin (20 µg ml^−1^) and neomycin (20 µgml^−1^), but sensitive to erythromycin (20 µg ml^−1^). It is also capable of forming effective nitrogen-fixing nodules on *Acacia saligna*. The predominant fatty acid of the type strain 1AS14I^T^ is summed feature 8 (C_18 : 1 _ω7*c*).

The type strain of *Rhizobium aouanii* sp. nov. 1AS14I^T^ (=DSM 113914^T^ = LMG 33206^T^), was isolated from root nodules of *Acacia saligna* used as trapping host in Borj cedria soil located in northeastern Tunisia. The genome size of strain 1AS14I^T^ is approximately 7.11 Mb with a G+C content of 60.93 mol%. The Genbank accession numbers for the 16S rRNA, *atpD*, *recA*, *glnII*, *gyrB*, *nodA* and *nodC* gene sequences are OM392036, OM429377, OM429400, OM429436, OM429451, OM429502 and OM429498, respectively. The GenBank draft genome accession number of strain 1AS14I^T^ is JBAMYD000000000.

## supplementary material

10.1099/ijsem.0.006515 Uncited Supplementary Material 1.

## Abbreviations

AM: agar medium
ANI: average nucleotide identity
dDDH: digital DNA–DNA hybridization
gsp: genospecies
ML: maximum-likelihood
YMA: yeast mannitol agar
