# Supplementary material for: Rhizobium aouanii sp. nov., efficient nodulating rhizobia isolated from Acacia saligna roots in Tunisia
Source: Int J Syst Evol Microbiol. 2024 Sep 5;74(9):006515. doi: 10.1099/ijsem.0.006515 (PMC11376454; doi:10.1099/ijsem.0.006515)
Supplement: Uncited Supplementary Material 1. [file ijsem-74-06515-s001.pdf]

Table S1. Average nucleotide Identity and digital DNA-DNA Hybridization (dDDH) values between the type strain of *Rhizobium aouanii* (1AS14I) and GenBank uncultured metagenome-derived genome entries.

Rhizobium isolates	Accession #	Genome size	ANI	dDDH	distance
Rhizobium sp. EaCA-2	PEKW01	4.8 Mb	80.0807	20.8	0.211
Rhizobium sp. UBA11406	DPXQ01	4.8 Mb	78.9725	20.1	0.219
Rhizobium sp. Bin_4_3	SSFH01	4.1 Mb	78.5928	19.9	0.2209
Rhizobium sp. MAG-91	JACIYZ01	5.5 Mb	80.1374	20.6	0.2128
Rhizobium sp. DR_1_3.37	JAHRXA01	4.5 Mb	79.0773	20.8	0.2108
Rhizobium sp. C31_metabat.bin.2	JAIBFB01	4.0 Mb	79.8394	20.9	0.2101
Rhizobium sp. BC_maxbin.001	JAIBEU01	5.2 Mb	79.3811	21.1	0.208
Rhizobium sp. LANL01D	JAHHDQ01	4.6 Mb	79.4844	20.1	0.2183
Rhizobium sp. LANL01E	JAHHDR01	6.0 Mb	78.6889	20.2	0.2176
Rhizobium sp. AG18	JAHRYZ01	4.4 Mb	79.7621	20.5	0.2148
Rhizobium sp. LE19-338.1A	JAPZRY01	4.2 Mb	79.3752	20.1	0.2182
Rhizobium sp. LE19-388.1D	JAPZUE01	5.1 Mb	79.4433	20.3	0.2167
Rhizobium sp. RYN_488	JAQGJP01	4.7 Mb	78.4117	20.2	0.2175
Rhizobium sp. RYN_489	JAQGJQ01	6.7 Mb	78.5081	20	0.2199
Rhizobium sp. B02.bin.8	JARXAQ01	4.1 Mb	78.9885	19.9	0.2211
Rhizobium sp. D19.bin.14	JARXAK01	4.0 Mb	79.3359	20.3	0.2165
Rhizobium sp. CZS25K_Bin3_fgap_89178_1	JAUCZD01	4.3 Mb	79.1845	20	0.22
Rhizobium sp. CCMP836_Bin11_fgap_56249_1	JAUCYR01	4.5 Mb	79.0965	19.9	0.2207
Rhizobium sp. CTOTU47978	DAMABX01	6.0 Mb	79.0306	20.3	0.217
Rhizobium sp. CTOTU47727	DALZSY01	4.8 Mb	81.8302	23	0.1903
Rhizobium sp. CTOTU48780	DAMBDH01	4.5 Mb	79.3946	20.5	0.2145
Rhizobium sp. CTOTU49051	DAMBMG01	6.6 Mb	79.0456	20.3	0.2159
Rhizobium sp. CTOTU49772	DAMCIK01	5.1 Mb	81.9339	22.9	0.1908
Rhizobium sp. CTOTU50516	DAMDJD01	4.9 Mb	78.8043	20.5	0.2146
Rhizobium sp. CTOTU50504	DAMDIR01	6.5 Mb	79.1818	20.3	0.2164
Rhizobium sp. CTOTU49707	DAMCFX01	5.6 Mb	78.7084	19.9	0.2206
Rhizobium sp. Salar_de_Ascotan_water_MAG_AWA9	JAXHOP01	4.9 Mb	79.9645	20.5	0.2144
Rhizobium sp. SMAG_U10590	DATGWU01	5.1 Mb	79.2208	20.8	0.2113
Rhizobium sp. CTOTU7690	DAIMJJ01	4.2 Mb	79.2894	20.5	0.2144
Rhizobium sp. P15_KT_PC_D28_E2_bin.10	JBBNAU01	6.5 Mb	81.0946	23.1	0.1891
Rhizobium sp. ERR2144888_bin.22_CONCOCT_v1.1_MAG	CAKZGA01	5.2 Mb	77.7601	21.2	0.2075
Rhizobium sp. ERR2144882_bin.0_CONCOCT_v1.1_MAG	CAKZGU01	5.4 Mb	79.4126	20.2	0.218
Rhizobium sp. SRR3901701_bin.43_CONCOCT_v1.1_MAG	CALCWM01	4.8 Mb	79.7613	20.5	0.2146
Rhizobium sp. SRR3330211_bin.104_CONCOCT_v1.1_MAG	CALKTX01	4.1 Mb	78.9538	20.8	0.2111
Rhizobium sp. SRR3901701_bin.3_CONCOCT_v1.1_MAG	CALLIZ01	7.9 Mb	80.5544	21.9	0.2007
Rhizobium sp. SRR9109403_bin.1_metaWRAP_v1.3_MAG	CAMFHE01	6.0 Mb	79.8315	20.7	0.2121
Rhizobium sp. SRR9109402_bin.13_metaWRAP_v1.3_MAG	CAMFHT01	4.1 Mb	78.8573	20.3	0.2161
Rhizobium sp. SRR9109406_bin.7_metaWRAP_v1.3_MAG	CAMFIE01	4.1 Mb	78.7737	19.9	0.2209
Rhizobium sp. SRR9109407_bin.14_metaWRAP_v1.3_MAG	CAMFIF01	4.5 Mb	79.2011	20.2	0.2179
Rhizobium sp. ERR9969061_bin.5_MetaWRAP_v1.3_MAG	CAUPWV01	4.6 Mb	79.1823	20.2	0.218

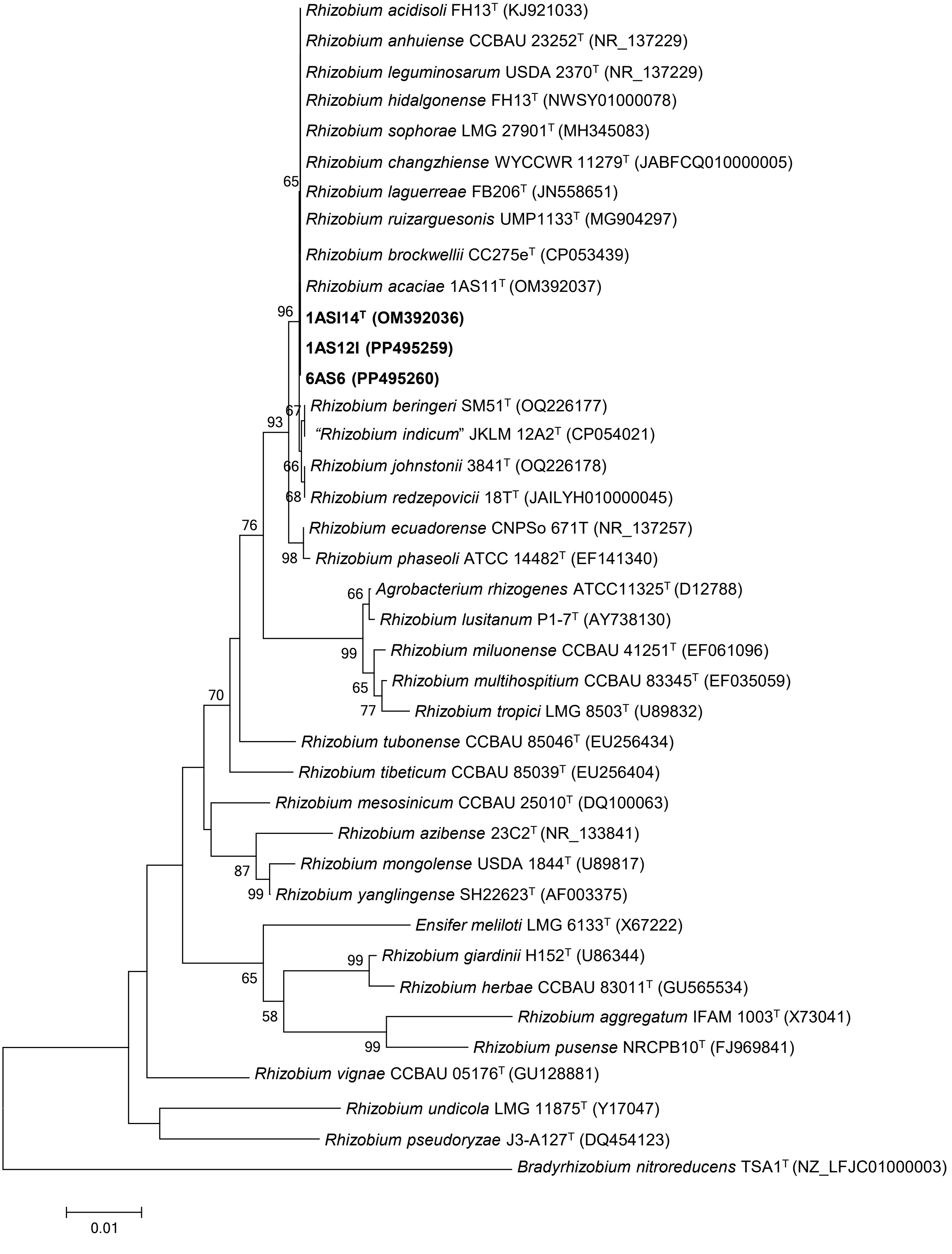

Fig. S1: Neighbor-Joining inferred phylogenetic tree based on rrs sequences (1215 nucleotides). The three novel strains are in bold. Bootstrap values ≥50 are indicated for each node (1000 replicates). After species name, the strain designation followed by the NCBI accession number of the sequence used. The scale indicates the number of substitutions per site.

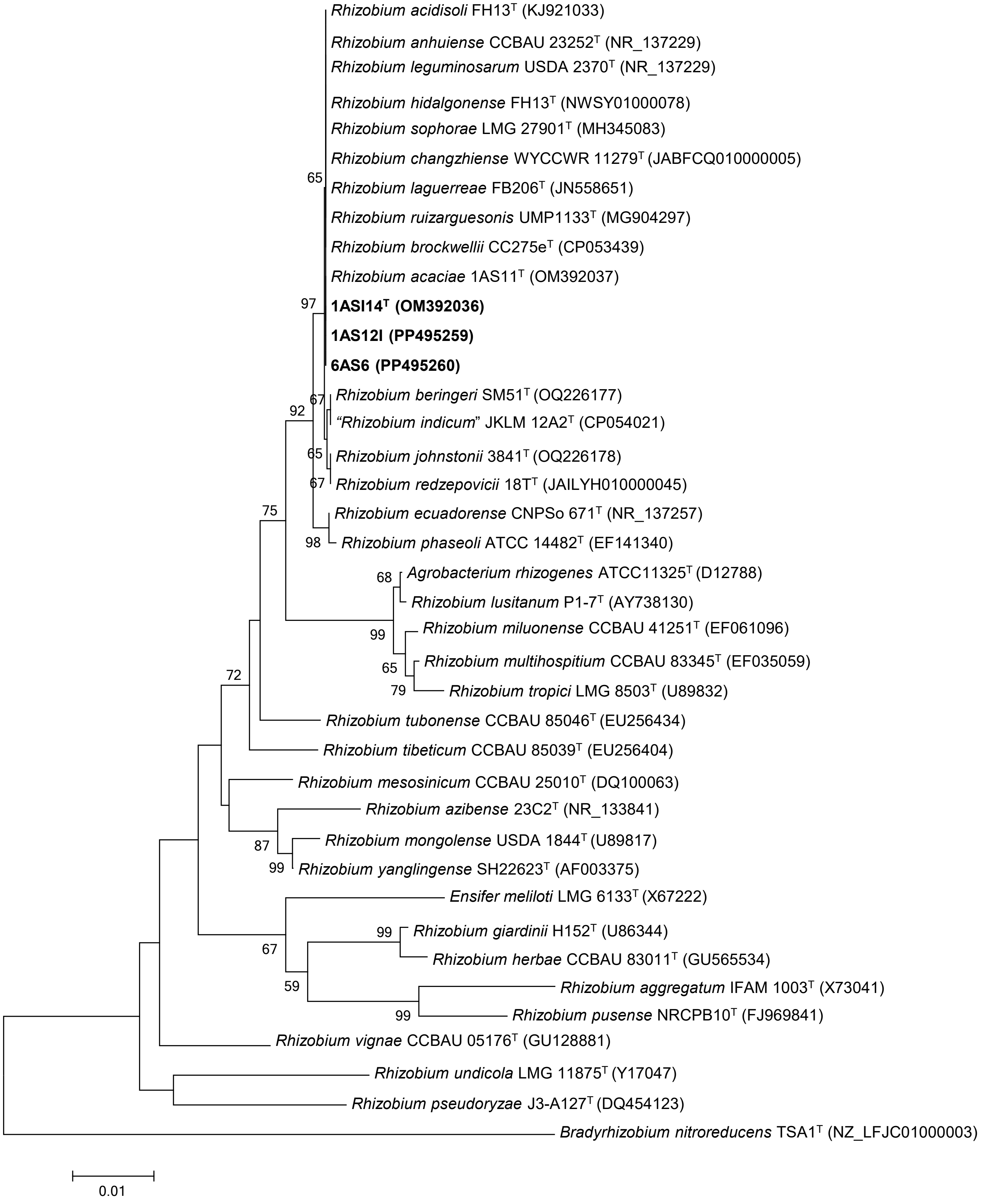

Fig. S2: Phylogenetic tree of rrs sequences (1215 nucleotides) based on the minimum evolution Method. The three novel strains are in bold. Bootstrap values ≥50 are indicated for each node (1000 replicates). After species name, the strain designation followed by the NCBI accession number of the sequence used. The scale indicates the number of substitutions per site.

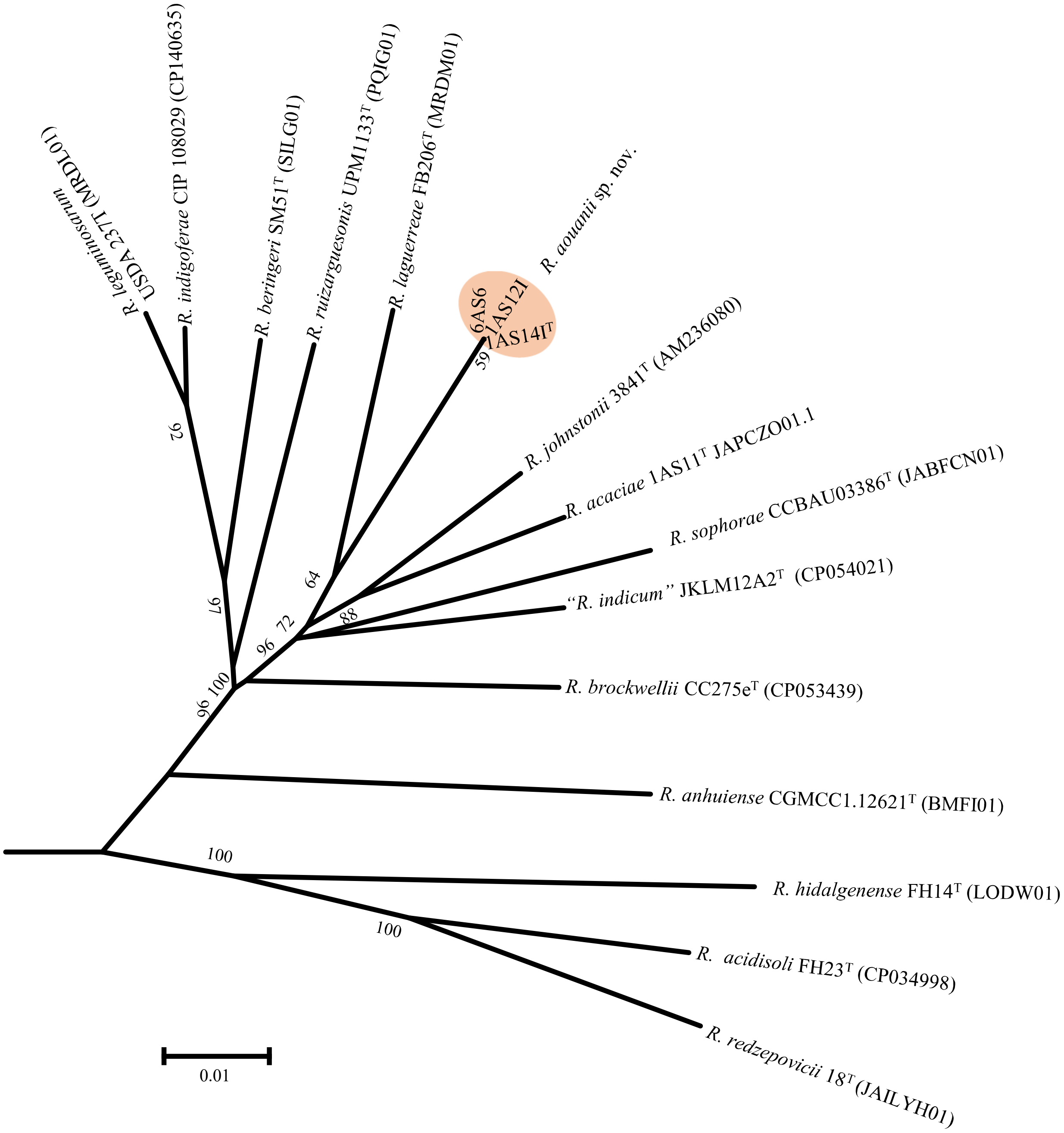

Fig. S3: TyGS phylogenomic tree inferred based on GBDP distances calculated from whole genome sequences of strains of *Rhizobium aouanii* and closest related species of the *R. leguminosarum* complex. The branch lengths are scaled in terms of GBDP distance formula d5. The numbers above branches are GBDP pseudo-bootstrap support values > 50 % from 100 replications, with an average branch support of 75.6 %.

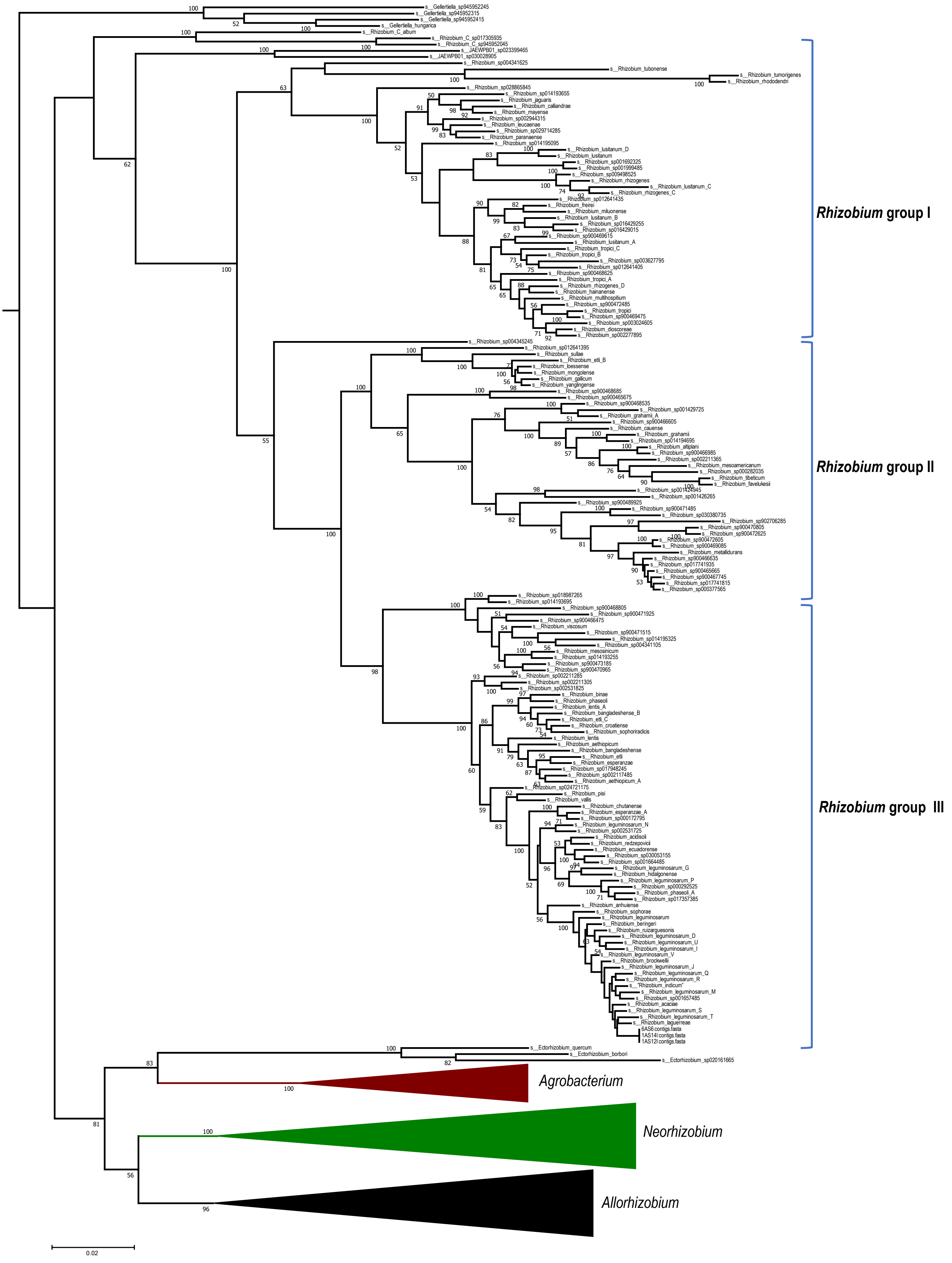

Fig. S4. Phylogenomic subtree of GTDB Bac120 protein set showing all the *Rhizobium* genomes in three distinct groupings relative to close genera *Ectorhizobium*, *Agrobacterium*, *Neorhizobium* and *Allorhizobium*. Tree generated using the classify_wf command and the most recent database (release220) of the Genome Taxonomy Database (GTDB-TK) pipeline v.2.4.0.

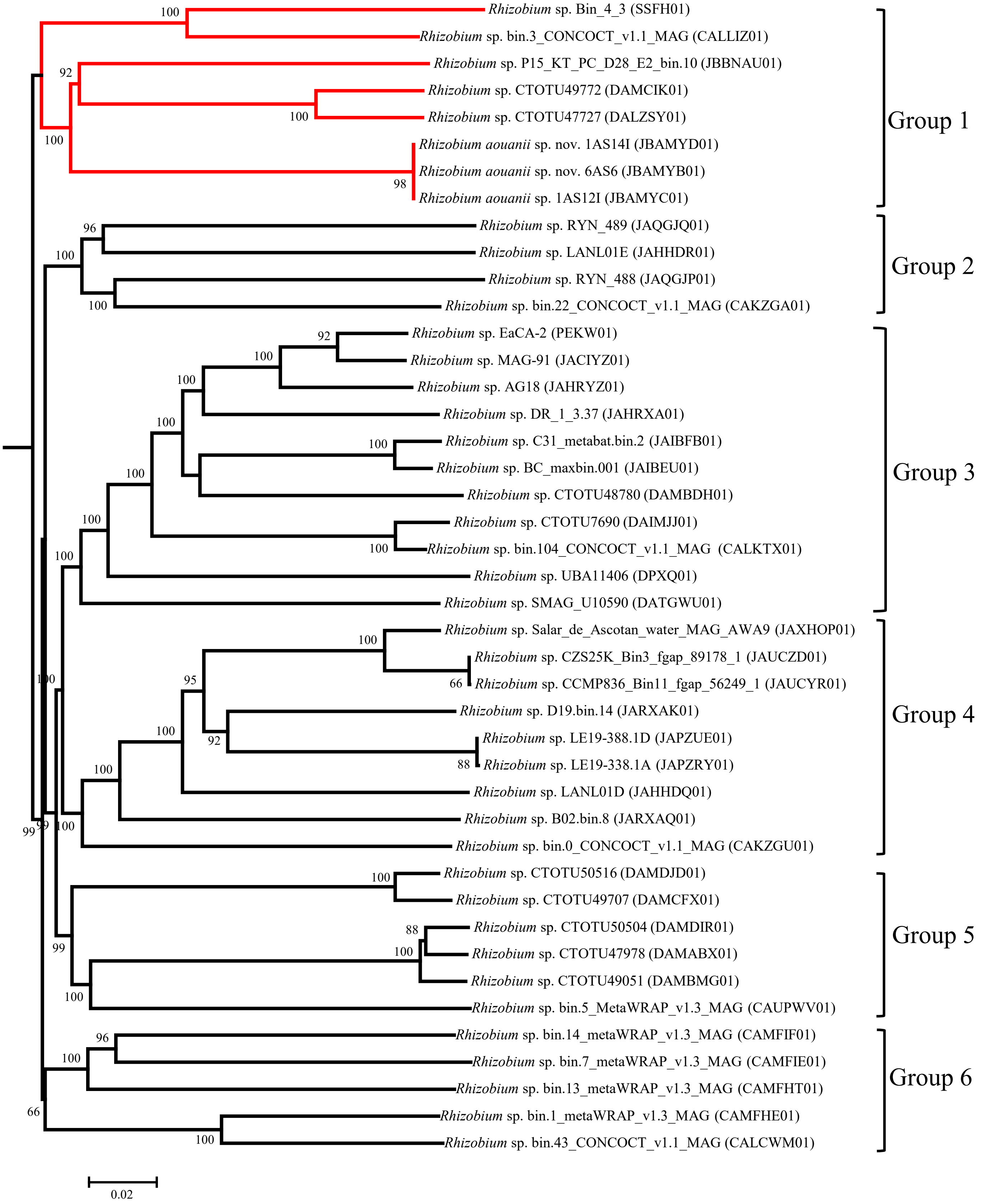

Fig. S5. Phylogenomic relationship of *Rhizobium aouanii* strains relative to Genbank uncultured metagenome-derived genome (MAG) sequences. TyGS-generated tree inferred from GBDP distances calculated from genome sequences. The branch lengths are scaled in terms of GBDP distance formula *d5*. The numbers above branches are GBDP pseudo-bootstrap support values > 60 % from 100 replications, with an average branch support of 91.6 %. The tree was rooted at the midpoint. Cluster in red colored branches shows strains of *R. aouanei* distantly grouping with five MAG sequences.
